# UNDERSTANDING VARIATIONS IN RELATIVE EFFECTIVENESS: A HEALTH PRODUCTION APPROACH

**DOI:** 10.1017/S0266462315000719

**Published:** 2015

**Authors:** Adrian Towse, Bengt Jonsson, Clare McGrath, Anne Mason, Ruth Puig-Peiro, Jorge Mestre-Ferrandiz, Michele Pistollato, Nancy Devlin

**Affiliations:** Office of Health Economicsatowse@ohe.org; Stockholm School of Economics; AstraZeneca; University of York; Servei Català de la Salut (CatSalut); Office of Health Economics

**Keywords:** Relative effectiveness, Drug evaluation, European Union

## Abstract

**Background:** Relative effectiveness has become a key concern of health policy. In Europe, this is because of the need for early information to guide reimbursement and funding decisions about new medical technologies. However, ways that effectiveness (does it work?) and efficacy (can it work?) might differ across health systems are poorly understood.

**Methods:** This study proposes an analytical framework, drawing on production function theory, to systematically identify and quantify the determinants of relative effectiveness and sources of variation between populations and healthcare systems. We consider how methods such as stochastic frontier analysis and data envelopment analysis using a Malmquist productivity index could in principle be used to generate evidence on, and improve understanding about, the sources of variation in relative effectiveness between countries and over time.

**Results:** Better evidence on factors driving relative effectiveness could: inform decisions on how to best use a new technology to maximum effectiveness; establish the need if any for follow-up post-launch studies, and provide evidence of the impact of new health technologies on outcomes in different healthcare systems.

**Conclusions:** The health production function approach for assessment of relative effectiveness is complementary to traditional experimental and observational studies, focusing on identifying, collecting, and analyzing data at the national level, enabling comparisons to take place. There is a strong case for exploring the use of this approach to better understand the impact of new medicines and devices for improvements in health outcomes.

Relative, or comparative, effectiveness has become a key health policy concern. Payers are increasingly interested in assessing the relative effectiveness of new healthcare technologies relative to standard care or other designated comparators (1). This reflects growing recognition of the need to understand how efficacy demonstrated in a randomized controlled trial (RCT) will translate into added benefit in routine clinical use in different healthcare settings. Relative effectiveness evidence is also important when assessing whether results in one jurisdiction can be applied elsewhere. Regulators want to use it in post-launch benefit risk assessments (2). In the case of the anti-obesity drug rimonabant, for example, such an assessment suggested both that relative effectiveness was poorer than relative efficacy and safety risks were higher than expected. The product was subsequently withdrawn by the European Medicines Agency (EMA) (3).

*Relative effectiveness* can be defined as “the extent to which an intervention does more good than harm compared with one or more alternative interventions under the usual circumstances of healthcare practice” (4). This contrasts with *relative efficacy*, which is a comparison “under ideal circumstances,” that is, “under clinical trial conditions” (5). Comparative effectiveness is very similar to relative effectiveness, and is defined as “comparing health outcomes and the clinical effectiveness, risks, and benefits of two or more medical treatments, services, and items” (6) which for Garber and Sox means “real world settings” (7). We focus on the European Union (EU) to explore the translation of relative efficacy into relative effectiveness in recognition of current efforts to develop EU-level approaches to assessment.

There appears to be a general view that relative efficacy of most therapies will be the same across Europe in most circumstances, although very few studies have tested that assumption (8). In the RE-LY trial, the relative efficacy of a new oral anticoagulant varied between countries even under RCT conditions, depending on the efficiency of warfarin management (9). Thus, even relative efficacy can differ between countries when healthcare practice varies.

How relative effectiveness might differ across health systems within the EU is poorly understood. Yet variations in relative effectiveness have implications for: (i) The extent to which national and regional pricing and reimbursement (P&R) decisions vary, and why. This will depend on whether decisions about patient access to medicines depend on considerations of relative effectiveness and, if so, the extent to which relative efficacy is accepted as a proxy for it. (ii) The desire to assess benefit–risk by the EMA beyond launch into the “product life cycle” in which expected benefits are compared with expected risks, based on evidence from actual use (10). (iii) The work of the European network for Health Technology Assessment (EUnetHTA) in particular Work Package 5 (WP5), to standardize methods and data requirements for relative effectiveness assessments across the Member States, including the applicability of evidence from one setting to another (11), following the recommendation of the High Level Pharmaceutical Forum in 2008. (iv) The EU drive to increase cross border health care in Europe: the implication is that citizens should obtain same health outcomes regardless of the country where they obtain a treatment. (v) The actions health systems can take to be sustainable and meet health needs in the face of economic downturn (12). In the United States, the purposes of conducting comparative effectiveness research include providing both the basis to improve care quality and to enable savings (13).

Health technology industries face a paradigm shift if they are to be paid and regulated on the basis of “does it work?” (effectiveness) instead of “can it work?” (efficacy) (14). Industry remuneration and regulatory benefit–risk assessment would also depend on the actual capacity, competence, or other ability of the healthcare system to provide access to and otherwise appropriately deliver the drug, that is, the effectiveness of the system, as well as the potential effectiveness of the drug. This has two consequences. First, there is an increased demand for studies to identify local impact. In the extreme, if a company has to plan to conduct different relative effectiveness studies for twenty-eight EU Member States post launch, the implications for development costs may be substantial, even if done in collaboration with healthcare systems. Second, evidence of relative effectiveness in routine use will need to be integrated into a range of decisions: not only decisions on reimbursement or regulatory status, but also decisions about any required changes to current care practices needed if the benefits of new technologies are to be optimized.

## METHODS

This study focuses on how we can identify whether and to what extent relative effectiveness differs across EU healthcare systems, and what data and methods are needed to study the impact of changes over time when new treatment options are introduced. We undertook a brief scoping review to identify approaches to measure relative effectiveness. We focused on literature reviews and empirical studies that distinguished between efficacy and effectiveness, that is, outcomes in clinical practice. We then explored the potential relevance of an analytical framework drawing on production function theory to consider how certain sets of inputs and processes yield specified outcomes. This approach can, in principle, provide a systematic way of identifying and quantifying the determinants of relative effectiveness, and sources of variation between populations and healthcare systems. We set out the elements of a multilevel model in this study. In an accompanying article (15), we use this model to categorize evidence on breast cancer to illustrate potential differences in the relative effectiveness of a new drug entering the market.

The production function approach is complementary to traditional experimental and observational studies, and focuses on identifying, collecting, and analyzing data at the national level for assessment of relative effectiveness. Our approach consisted of four steps. First, we set out an analytical framework. The health production function approach uses health as the output of interest. This reflects the EU Directive on cross border care which forms the basis for the coordination of health technology assessment (HTA) and relative effectiveness assessment in Europe (16). Relative effectiveness is the basis for assessing therapeutic added value and cost-effectiveness at the national level. What determines value is the *absolute* difference in effectiveness, which translates for example into the number of QALYs gained. Second, we classify inputs (or “factors”) into three main levels: patient, provider, and the healthcare environment or system. The relative effectiveness of a drug is the additional net output (health) achieved by adding a new drug to usual care or substituting it for another treatment. Third, the health production function provides an approach to organize the data and an analytical framework to examine the differences in relative effectiveness across jurisdictions. We briefly outline the types of model that might be appropriate and consider its role in relation to the increasing demand for observational studies of the impact of the use of drugs in practice. Lastly, we consider the policy implications of our analysis.

## RESULTS

### Understanding the Reasons for Differences in Relative Effectiveness

Eichler et al. (1) set out the methodological options and challenges in measuring relative efficacy. Their subsequent comprehensive review (5) identified two reasons for an “efficacy-effectiveness gap”: biological (including genetics, age, sex, co-morbidity and baseline severity of disease) and behavioral (e.g., variation in adherence to the treatment regimen). Economists would add that any understanding of a potential “efficacy-effectiveness gap” requires translating trial outcomes into long-term health outcomes. However other factors will also play a role, notably the health system. For example, the health system and the environment impact on disease severity and co-morbidity. Adherence is not only behavioral, but also depends on the organization and resources used for managing adherence, and thus the efficiency of the healthcare system. The selection of patients for treatment and the management of patients over time will depend on incentives, organization and resources in the healthcare system. These elements may differ across jurisdictions and will impact on long-term outcomes.

### Estimating Relative Effectiveness: Empirical Methods

Currently relative effectiveness, as opposed to relative efficacy, is treated in the literature and assessed by HTA and reimbursement agencies in one of three ways: (i) It is assumed to be the same as relative efficacy, and any potential “efficacy-effectiveness gap” is ignored, that is, modelling is based on clinical trial data. (ii) It is estimated using decision analytical modelling. However, this technique is typically used to project longer term health outcomes from short term or intermediate efficacy measures, or to make indirect comparisons, rather than to model the impact of the health system on the translation of the efficacy of a technology into outcomes in routine care. An intermediate case is when the relative risk reduction from a trial is applied to the absolute risk level for different groups of patients in different countries (17). However, this approach does not take into account other factors that may influence relative effectiveness, for example differences in adherence between countries. (iii) Observational studies or pragmatic clinical trials are proposed as methods to explore outcomes in routine clinical practice within a healthcare system (17–19). The number of observational studies being undertaken within health systems is increasing, but they can be time consuming and expensive unless relevant data are already routinely captured by a healthcare system. Analysis can be complex and results open to differing interpretations, although methods to tackle confounding and other issues are improving. Pragmatic trials also can be time consuming and expensive unless infrastructure investment is put in place and recruitment streamlined.

None of these approaches are routinely used to detect and explain differences in relative effectiveness across health systems. A review of the literature (8) found no observational studies investigating variations in relative effectiveness across Member States, although there were safety studies and studies comparing other aspects of care across several Member States. It is, therefore, not possible to address the question of differences in relative effectiveness on the basis of current evidence.

One route forward is for more observational studies or pragmatic clinical trials to be conducted across Member States. Given the cost and complexity of conducting them, however, it would make sense to explore another approach which can use existing data, and could be used to help identify whether differences in relative effectiveness were likely to be of an order to merit further study.

### The Conceptual Framework: A Health Production Function Model of the Determinants of Relative Effectiveness

The relative effectiveness of a new technology is likely to depend on how it is combined with other health inputs, and a range of other variables that influence the overall relationship between inputs and health outcomes in the underlying “health production function.”

Assuming the comparator treatment is one of the inputs in the initial health production function, then relative effectiveness is the change that results when the new treatment is introduced relative to that obtained from the existing (or usual) treatment (and other inputs). In this context, “usual care” in the health system will be a key determinant of the relative effectiveness of any new treatment. The underlying research questions are: if a medicine for, say, breast cancer is granted Market Authorization by the EMA, indicating that the new technology is available in all EU countries, do we expect that its relative effectiveness will be different across them? If so, why?

One way to investigate influences on relative effectiveness is to treat this analytically as a multilevel model, separating the different levels of influence. Influences may operate on one or more of three main levels: patient, provider, and the healthcare environment and system (see Table 1): (i) “Individual” patient level factors refer to the characteristics of the patients being treated, including their demographic, genetic, and clinical characteristics. Not only may the patient mix gaining access to the technology differ but underlying population health may also differ between health systems. The same technology in the same setting may, therefore, produce different health effects depending on patient characteristics. Even if *relative* efficacy is the same across two health systems, if baseline risk differs then size of the *absolute* incremental effect measured by relative effectiveness may differ. (ii) The characteristics of the providers. The same technology may produce different health effects on patients with similar characteristics depending on how it is used and combined with other technologies in clinical practice (e.g., screening policy, or treatment setting; see Table 1). Clinical practice may vary in other ways that influence treatment outcomes (both from new technologies and comparators) for example due to economic and other incentives for providers. (iii) Healthcare environment and system factors. The availability or otherwise of technologies within the healthcare system and how technologies are used (and hence the health effects on patients) (i.e., “comparators” in Table 1) is often driven by P&R policies and clinical guidelines from regional or national decision makers. Waiting times for referral and then access to specialist care also vary between (and within) countries.

**Table 1.** Overview of Factors That May Influence the Relative Effectiveness of a Medicine or Other Technology

Influence level	Category	Variables
Individual / patient level factors	Demographic characteristics	Age; gender; socioeconomic status; education; insurance status; employment status; lifestyle factors (e.g., smoking status)
	Clinical characteristics	Disease severity, including disease stage at diagnosis; co-morbidity; procedures received; general health and life expectancy; genetic type
	Other characteristics	Compliance /concordance; health literacy/awareness
Provider level factors	Provider characteristics	Specialization; private/public status; teaching status; clinical practice (e.g. hospital policy on use of off-label drugs); capital / labor availability (e.g. physician skills, CT scanner); patient selection.
Environment/health care system level factors	Population health	Lifestyles, mortality rates, life expectancy, disease prevalence; valuation of outcomes (e.g. EQ-5D values)
	National / regional guidelines / regulations	Clinical guidelines; national service frameworks/national plans; legal framework (e.g. funding for some drugs / services may be mandatory)
	Service delivery and organization	Screening, treatment settings, provision of palliative care
	Access issues (local regional/national)	Pricing and reimbursement policies; insurance and co-payment policies; waiting time targets; comparator drug(s) available
	Economy	GDP; % GDP spent on health care; % private funding for health care

Some determinants of outcome operate and interact at several levels. Five-year survival rate for breast cancer may differ across countries due (in part) to differences in all-cause mortality rates. These may be due to individual-level effects (e.g., smoking status), to environmental effects (e.g., taxes on cigarettes), or to health system public health measures restricting smoking. The key is to identify factors driving outcomes and ensure they are included, but only once. In this example, the variable for smoking would be most appropriately classified and included in the analysis as an individual level effect. In the accompanying article (15), we provide an overview of factors affecting breast cancer outcomes identified from a literature review, grouping them according to the multilevel approach: “individual level,” “provider level,” and “environment and healthcare system level” set out in Table 1. The results are set out in Table 2 of Puig-Peiro et al. (15).

### The Malmquist DEA Model for Measuring Relative Effectiveness

The theory and methods for estimating health production functions have developed rapidly over the past 2 decades. Important contributions include the development of econometric approaches such as the stochastic frontier approach (20;21), and also data envelopment analysis (DEA) (22;23), including the use of Malmquist indexes which allow use of multiple input, multiple output models without prices. The latter is particularly important in a European healthcare perspective, where prices vary between countries, and often are not possible to observe and calculate in public healthcare systems. In addition, for some inputs to the health production function, such as an individual's time and effort used for prevention and treatment prices are not directly observable, as is also the case for the output health. Using index numbers that include prices for measurement of changes in total factor productivity, that is, relative effectiveness is not an option.

A further advantage of the Malmquist approach is that, once the production technology is estimated, either as a production frontier or its dual the cost frontier, one can decompose the change in two component parts: efficiency change and technical change. The methodology proposed by Färe et al. (1994) (24) uses DEA to calculate distance functions to produce the Malmquist total factor productivity index, and then decomposes this into technical and efficiency components respectively. This decomposition is important because the change in relative effectiveness in a given countries is made up by a combination of changes in the frontier, and how the country is moving in relation to the frontier.

**Figure 1.**
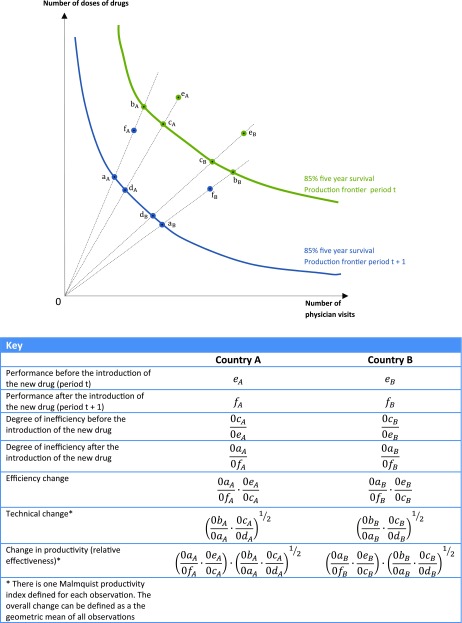
Illustration of the Malmquist Index applied to the identification of possible variations in the relative effectiveness of breast cancer treatments.

Figure 1 illustrates how a Malmquist DEA model could help identify and explain potential cross-country variations in the relative effectiveness of the introduction of a new treatment for breast cancer treatment. It can be used to study and decompose the productivity changes both in terms of technical change (best practice change, shown by a shift in the frontier) and technical efficiency change (countries moving closer to the frontier), that is, to understand how the introduction of a new technology changes outcomes, and hence to establish its relative effectiveness. This is essential for the design of policies aimed at improving relative effectiveness.

For illustrative purposes, we assume just two inputs, but in practice the estimation of these models can include multiple inputs and patient attributes such as those set out in Table 1 that will impact on the patient outcomes. The horizontal axis measures the number of “physician visits” as a proxy for the intensity of monitoring and nondrug treatment, and the vertical axis measures the number of doses of “drugs” as a proxy for the intensity of drug treatment. We could also have used for example the share of visits to a specialist on the horizontal axis and an index of “vintage,” that is, year of introduction, for drugs. For illustration, we show the various combinations of the two variables that yield an 85 percent 5-year survival rate. The Production Frontier in Figure 1 is data-driven: it is the envelope of the most efficient data points observed from all countries in the group. We compare the average performance of two of the countries, A and B.

In period t, before the introduction of the new drug, efficiency is not the same in the two countries: e_A_ is closer to the production frontier in period t than is e_B_. They use different combinations of input factors, which may reflect differences in relative prices between drugs and physician visits in the two countries. The distance function 0c/0e represents the technical inefficiency in country A and B respectively in period t, that is, how much the use of drugs and visits could have been reduced if they had applied the best practice use suggested by the production frontier at period t.

When a new drug or procedure is introduced in period t+1, the production frontier shifts inward, indicating an improvement in technical efficiency to produce the same level of health outcome with fewer of each of the inputs. The frontier may not change by the same proportion for both countries suggesting that one country may increase its efficiency in a larger proportion than the other after the introduction of the new drug.

The distance functions 0a/0f shows the new degree of inefficiency, that is, actual performance compared with best practice on the radial from the origin for the two countries, respectively.

For each observation, the change from period t to t+1 in efficiency can be computed as the ratio between the two distance functions, and the technical change as the geometric mean of the product of two ratios. These are defined in the key to Figure 1. The total productivity change, or change in relative effectiveness is the product of the two ratios. All observations can be aggregated to an overall productivity change, using the geometric mean.

The optimal mix of drugs and physician visits that would occur once the new drug was available along the production frontier in period t+1 depends on their relative prices and can be studied separately.

We show, in Online Supplementary File 1, the potential use of this approach with a very simple calculation and illustration of the technical efficiency with which combinations of breast cancer screening and treatment with trastuzumab are used in seven European countries.

### Strengths and Weaknesses of the Approach

Using such an approach to empirically identify and explain differences in relative effectiveness may encounter some challenges in practice. For example, it would require data on all relevant factors to be available in a manner that facilitates cross-country comparisons. Furthermore, depending on the specific factors identified as relevant to understanding relative effectiveness in a specific disease area, relationships between explanatory variables (e.g., adherence may depend on socioeconomic status) can confound identification of their relationship to outcomes. However, such challenges are common to many studies and do not preclude useful and valuable research being undertaken.

Valid and reliable price data for (non-drug) inputs and outputs are generally not available, so techniques to estimate cross- and time-series variations in the relationship between inputs and outputs without price data, such as the approach illustrated in Figure 1, are an important step forward. They have been extensively used to understand the relative efficiency of providers (usually hospitals) within a single healthcare system and to compare the performance of healthcare systems; see Hollingsworth and Wildman (25), Greene (26), and the EUROHOPE project (27). Jacobs et al. (28) and Coelli et al. (29) provide a comprehensive review of the main econometric approaches for modelling health production functions.

Questions for the decision makers, based on such an analysis, should include: (i) whether a country can get closer to best practice and improve relative effectiveness; (ii) whether the likelihood of gaps between relative efficacy and relative effectiveness mean that post-launch studies are required; and (iii) whether potential differences between countries A and B mean that separate post-launch studies are needed in each.

We are not proposing econometric and economic modeling as an *alternative* to a prospective observational study or pragmatic trial to understand differences across healthcare systems in the relative effectiveness of a technology. Rather, we see this analytical tool as complementary: one that can use existing data (including data already available from post-launch studies) to explore the extent to which the performance of the same technology differs, or might be expected to differ, across settings as a result of differences in populations and healthcare systems.

### Policy Implications

There are *prima facie* reasons why we might expect some convergence in relative effectiveness across the EU over time: the diffusion of medical technology is becoming global and there is a flow of information about best practice. It is also possible to observe some convergence of healthcare systems in terms of financing, organization, and performance. Yet our findings suggest it is reasonable at the moment to expect meaningful and persistent variations in relative effectiveness between and within countries. Although the literature indicates that current understanding of what might be important is limited, each case is likely to be driven by a few key factors. The health production function is a tool for identifying these at an early stage and making predictions of their likely quantitative impact. However, it can also serve as a basis for targeting observational studies and pragmatic clinical trials post launch, to test predictions and gain further insight into how to best make use of the new technology, including the potential advantages and disadvantages of using coverage with evidence development and risk sharing arrangements to assess and reward relative performance.

There are four broad policy implications from our analysis. First, at a time when we are seeing that countries increasingly look at P&R decisions and HTA assessments in other countries, the debate around the transferability of relative effectiveness evidence for P&R decisions could be better informed by an understanding of what factors in a healthcare system are likely in practice to impact on the incremental performance of a technology.

Second, the increasing demand for post-launch studies to address uncertainty about relative effectiveness of drugs needs to be put in the context of such an understanding. Studies are expensive and complex and unnecessary duplication of effort could waste time and resources and delay patient access to effective treatments. A collaborative approach between industry and governments (and in the EU across the twenty-eight Member States) is required to: (i) Limit the number of extra studies. An improved understanding of the drivers of relative effectiveness can help prioritize these studies. They can be limited to those needed to explore key expected differences between health systems that could impact patient outcomes. (ii) Collect and provide access to necessary data on the relevant contextual factors. At present, access to routine data (for example from registers) is often restricted. In Sweden, a public inquiry described the registers as “a gold mine” for collecting evidence to improve health care and health. Such registers are, however, often oriented to answering specific clinical questions and need to be augmented with a wider set of relevant factors and outcomes for the analysis of relative effectiveness.

Third, where differences in relative effectiveness reflect variation in the efficiency of healthcare systems, good practice can be identified and shared. Decision makers in poorly performing health systems, who are responsible for the procurement of new technologies and for managing healthcare providers, can learn from good practice elsewhere. Relative effectiveness is the outcome of an interaction between a technology, patients, and the health system (including the providers). The role of the latter and how to improve efficiency is often missing from debates such as that about developing an EU assessment of relative effectiveness. Failure to improve health system performance risks wasted resources or poorer health outcomes.

Fourth, if industry remuneration and regulatory benefit–risk assessment increasingly depend on the actual effectiveness of the healthcare system in using a drug, as opposed to relative efficacy at launch, then we may see an increasing use of “pay for performance” and risk sharing agreements. Such agreements are, in effect, seeking to estimate the health production function and link payment in some way to the use or outcome. Without an understanding of the contextual factors, there is a danger of companies taking the “risk” for factors that are really the responsibility of the health system and *vice versa*. The potential for using post-launch follow-up studies to reduce the time and cost of drug development before market authorization in some areas (cancer being an example where fast access is important and risks can be managed) needs to be informed by an understanding of the key factors driving relative effectiveness.

## CONCLUSIONS

We conclude that the step from relative efficacy to relative effectiveness can be substantial and is likely to vary across health systems. Relative efficacy is a function of the attributes of the technology and target patient population. Relative effectiveness additionally depends on the performance of the healthcare delivery system and wider environmental factors. The potential for differences in relative effectiveness across EU Member State health systems gives rise to several public policy issues. The health production function approach is complementary to traditional experimental and observational studies, focusing on identifying, collecting, and analyzing data at the national level for assessment of relative effectiveness, enabling comparisons to take place. There is a strong case for exploring the use of this approach to enable the differences to be estimated and addressed and any requirements for post-launch observational or experimental studies to be targeted.

## Supplementary material

For supplementary material accompanying this paper visit http://dx.doi.org/10.1017/S0266462315000719. click here to view supplementary material

## CONFLICTS OF INTEREST

The authors report no conflict of interest.
