# Supplementary material for: UNDERSTANDING VARIATIONS IN RELATIVE EFFECTIVENESS: A HEALTH PRODUCTION APPROACH
Source: Int J Technol Assess Health Care. 2015;31(6):363–70. doi: 10.1017/S0266462315000719 (PMC4824958; doi:10.1017/S0266462315000719)

**Online Supplementary File 1**

**Illustrative simple example of the use of a health production frontier**

We show the potential use of the health production frontier approach with some easy to follow illustrative calculations of the technical efficiency with which seven European countries use combinations of two inputs, breast cancer screening and trastuzumab treatments, to achieve the output of breast cancer survival.

The example is highly simplified because we assume that the output is driven only by combinations of two inputs. We also assume constant returns to scale ―an increase of all the inputs at once in a given proportion increases the output exactly in the same proportion― and strong disposability of inputs ―it is possible to produce at least the same amount of output with more inputs. This could mean that not all additional inputs necessarily increase outputs (for example, if trastuzumab is given to a woman who is not HER2 positive it will not increase her survival prospects).

Table 1 below sets out for each country and Figure 1 illustrates the position of each country in relation to the frontier. The frontier is defined by the countries achieving an input efficiency index of 1. These are the Denmark, Germany and the UK. We can see that, in this simple illustration, Sweden, for example, appears to be technically inefficient. It uses similar amounts of screening per unit of survival as the UK, but more trastuzumab per unit of survival.


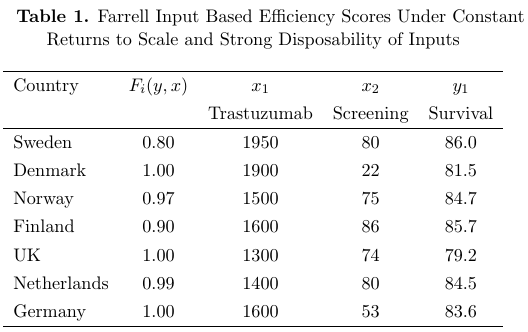


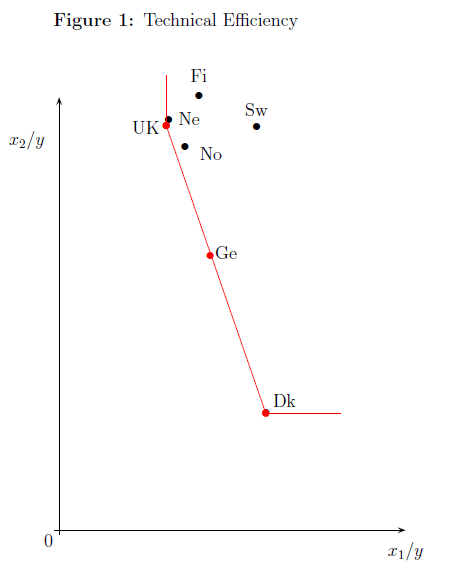

Supplement: Supplementary file 1 [file S0266462315000719sup001.docx]
